# CD25^*+*^ B-1a Cells Express *Aicda*

**DOI:** 10.3389/fimmu.2017.00672

**Published:** 2017-06-20

**Authors:** Hiroaki Kaku, Nichol E. Holodick, Joseph R. Tumang, Thomas L. Rothstein

**Affiliations:** ^1^Center for Oncology and Cell Biology, The Feinstein Institute for Medical Research, Manhasset, NY, United States; ^2^Compass Therapeutics LLC, Cambridge, MA, United States; ^3^Department of Medicine, The Hofstra Northwell School of Medicine, Manhasset, NY, United States; ^4^Department of Molecular Medicine, The Hofstra Northwell School of Medicine, Manhasset, NY, United States

**Keywords:** AID, B-1a cells, CD25, B-1 cell subset, peritoneal cavity

## Abstract

B-1a cells are innate-like B-lymphocytes producing natural antibodies. Activation-induced cytidine deaminase (AID), a product of the *Aicda* gene, plays a central role in class-switch recombination and somatic hypermutation in B cells. Although a role for *Aicda* in B-1a cells has been suggested on the basis of experiments with knock out (KO) mice, whether B-1a cells express *Aicda*, and if so, which B-1a cell subpopulation expresses *Aicda*, remains unknown. Here, we demonstrate that B-1 cells express *Aicda*, but at a level below that expressed by germinal center (GC) B cells. We previously reported that B-1a cells can be subdivided based on CD25 expression. We show here that B-1a cell *Aicda* expression is concentrated in the CD25^+^ B-1a cell subpopulation. These results suggest the possibility that previous studies of memory B cells identified on the basis of *Aicda* expression may have inadvertently included an unknown number of CD25^+^ B-1a cells. Although B-1a cells develop normally in the absence of *Aicda*, a competitive reconstitution assay reveals enhanced vigor for AID KO B-1a cell bone marrow (BM) progenitors, as compared with wild-type BM B-1 cell progenitors. These results suggest that AID inhibits the development of B-1a cells from BM B-1 cell progenitors in a competitive environment.

## Introduction

B-1 cells are innate-like B-lymphocytes that spontaneously and constitutively produce natural antibodies, which provide immediate protection against infection and rapid removal of dying cell debris (1, 2). Mouse B-1 cells are distinguished from B-2 cells both by phenotype and by function (3). Phenotypically, B-1 cells are characterized as IgM^hi^, IgD^lo^, B220^lo^, CD23^−^, and CD43^+^ (and CD11b^+^ in the peritoneal cavity) (3). B-1 cells are either CD5^+^ (B-1a) or CD5^−^ (B-1b). Recent studies have shown B-1 cells can be further subdivided based on the expression of CD25 (4), CD73 (5), PD-L2 (CD273) (6–9), or ENPP1 (PC1) (10), suggesting different roles for each subpopulation.

Activation-induced cytidine deaminase (AID) plays a central role in class-switch recombination and somatic hypermutation (11). AID is expressed abundantly in germinal center (GC) B cells (12) and at a low level in immature B cells (13–15). It was recently reported that B-1 cells accumulate immunoglobulin somatic hypermutation and increase class switching from 1 week of age up to 6 months of age, and these changes are diminished in the absence of AID (16). Nonetheless, AID expression in B-1 cells has not been documented and is yet to be directly addressed, raising the possibility that B-1 cell changes in AID KO mice may represent indirect effects.

We previously found that expression of CD25 on B-1a cells is activation dependent and these CD25^+^ B-1a cells express leukemia inhibitory factor receptor as well as increased levels of activated STAT3 as compared to CD25^−^ B-1a cells (4). We explored the possibility that B-1 cells express AID and found that AID is expressed in B-1a cells and that this expression is concentrated in the activated, CD25^+^ B-1a cell pool.

## Materials and Methods

### Mice

Male BALB/c-ByJ and C57BL/6 mice were obtained from The Jackson Laboratory at 6–8 weeks of age. CB17-SCID or CB17 mice of 6–8 weeks of age were obtained from Taconic. AID KO mice on a BALB/c background were obtained from Dr. Michel Nussenzweig with Dr. Tasuku Honjo’s permission. All mice were used for experimentation at 8–14 weeks of age. All studies were approved by the Institutional Animal Care and Use Committee at the Feinstein Institute for Medical Research. Mice were cared for and handled in accordance with the National Institutes of Health and institutional guidelines.

### Cell Purification and Flow Cytometry

Peritoneal washout cells and splenocytes were obtained from 8- to 14-week-old wild-type (WT) or AID knock out (KO) mice and were stained with fluorescence-labeled antibodies to B220, CD5, CD25, CD23, and GL-7 and with peanut agglutinin (PNA). B-cell populations (peritoneal B-1a cells: B220^lo^/CD5^+^, peritoneal CD25^+^ B-1a cells: B220^lo^/CD5^+^CD25^+^, peritoneal CD25^−^ B-1a cells: B220^lo^/CD5^+^CD25^−^, splenic B2 cells: B220^hi^CD5^−^CD23^+^, or GC B cells: B220^+^/GL-7^+^/PNA^high^) were isolated using the Influx cell sorter (BD Biosciences). Post-sort, reanalysis of the B-cell populations showed them to be ≥98% pure. Cells were blocked with rat anti-mouse CD16/CD32 antibody (clone 2.4G2), stained with immunofluorescent antibodies, and then analyzed on a FACSCalibur flow cytometer (BD Biosciences) with appropriate gating. Images were constructed with FlowJo 6.0 software (Tree Star). PE-conjugated rat anti-mouse CD25 (clone PC61) was obtained from BD Pharmingen. The following antibodies were obtained from Biolegend: perCP-Cy5.5-conjugated rat anti-mouse CD45R/B220 (clone RA3-6B2); Alexa 647-conjugated rat anti-mouse CD5 (clone 53-7.3); and Alexa 647-conjugated rat anti-mouse GL-7. FITC-PNA was obtained from Sigma. CD23-PE-Cy7 (clone 2G8) was obtained from Abcam.

### Gene Expression

Gene expression was assayed by real-time PCR as previously described (17). Briefly, RNA was prepared from B cells using the RNeasy mini kit (Qiagen), according to the manufacturer’s instructions. cDNA was prepared using avian myeloblastosis virus reverse transcriptase (Bio-Rad). Gene expression was then measured by real-time PCR using iTaq SYBR Green (Bio-Rad) and normalized with β_2_-microglobulin. The following primer sets were used: β_2_-microglobulin (F-CCCGCCTCACATTGAAATCC/R-GCGTATGTATCAGTCTCAGTGG); AID (AGAAAGTCACGCTGGAGACC/CTCCTCTTCACCACGTAGCA). Gene expression was also measured by real-time PCR using TaqMan chemistry. Primer and probe sets were obtained from Applied Biosystems for Aicda (Mm01184115_m1) and β-actin, which was used for normalization.

### Adoptive Transfer

Bone marrow (BM) was obtained from 2-month-old BALB/c-ByJ (IgM^a^) mice and 2-month-old CB17 mice (IgM^b^). BM B-1a cell progenitors (lineage negative, CD19^+^B220^lo/−^AA4.1^+^) were sort-purified using the Influx cell sorter (BD Biosciences), washed twice in 1× PBS, resuspended in 1× PBS, and then injected (i.v.) into recipient CB17-SCID mice at 0.6 × 10^6^ cells per mouse in 0.2 ml. Recipient mice were not irradiated prior to transfer. Serum samples, spleens, and peritoneal washout cells were collected from euthanized CB17-SCID recipients 6 weeks post transfer.

### Statistics

Comparisons were conducted between WT and AID KO mice using Graphpad Prism 6.0 with two-tailed tests as indicated in the figure legends.

## Results

### B-1a Cells Express *Aicda* and Gene Expression Is Restricted to the CD25^+^ B-1a Cell Subset

**Figure 1:**
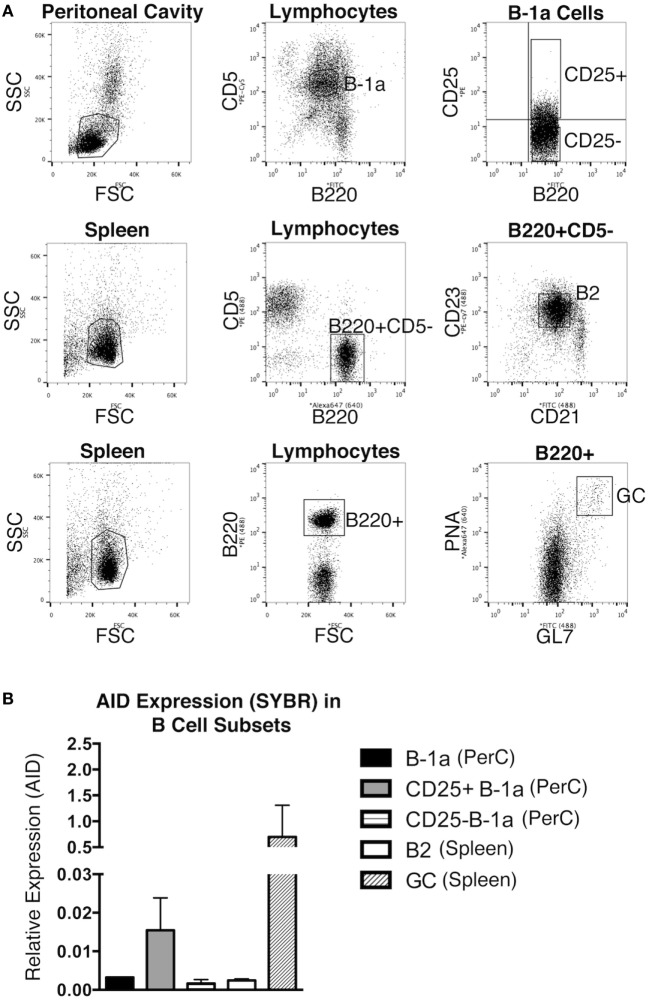
*Aicda* gene expression in B cells. Peritoneal washout cells and spleen cells were obtained from 3-month-old BALB/c-ByJ mice, immunofluorescently stained, and sorted for peritoneal B-1a (B220^lo^CD5^+^), CD25^+^ B-1a (B220^lo^CD5^+^CD25^+^), CD25^−^ B-1a (B220^lo^CD5^+^CD25^−^), splenic B2 (B220^+^CD5^−^CD23^+^), and germinal center (GC, B220^+^/GL-7^+^/PNA^high^) cells. The sorting strategy for these populations is shown in **(A)**. RNA was prepared from each sort-purified B cell subset and reverse transcribed. **(A)** The level of *Acida* relative to β_2_-microglobulin was determined by real-time PCR (SYBR Green) with the primers described in Section “Materials and Methods.” The means of three independent experiments are shown in **(B)**, along with lines indicating SEMs.

The expression level of *Aicda* was evaluated in sort-purified peritoneal B-1a cells, peritoneal CD25^+^ B-1a cells (4), peritoneal CD25^−^ B-1a cells, splenic B2 cells, and GC B cells from unmanipulated mice. The sorting strategy for isolating these populations is shown in Figure 1A. GC B cells displayed a high level of *Aicda* expression, which is consistent with previous reports (12), in contrast to splenic B-2 cells that expressed very little *Aicda*. We found that peritoneal B-1a cells expressed more *Aicda* than that by splenic B-2 cells, but less than that by GC B cells (Figure 1B). We then examined CD25^+^ B-1a cells in comparison to CD25^−^ B-1a cells and found that CD25^+^ B-1a cells demonstrated a higher level of *Aicda* expression than did CD25^−^ B-1a cells, total B-1a cells, and splenic B-2 cells, although this was still less than the level expressed by GC B cells. These results were confirmed using Taqman primers and probe (Figure S1 in Supplementary Material). Peritoneal CD25^+^ B-1a cells from C57BL/6 mice were also found to express *Aicda* in greater amounts than that by CD25^−^ B-1a cells (Figure S2 in Supplementary Material). The mean level of *Aicda* expression in BALB/c CD25^+^ B-1a cells was 18-fold more than that of splenic B-2 cells but 40-fold less than that of GC B cells. Thus, B-1a cells, especially CD25^+^ B-1a cells, express *Aicda*.

### The Number of CD25^+^ B-1a Cells Is Unchanged in AID KO

**Figure 2:**
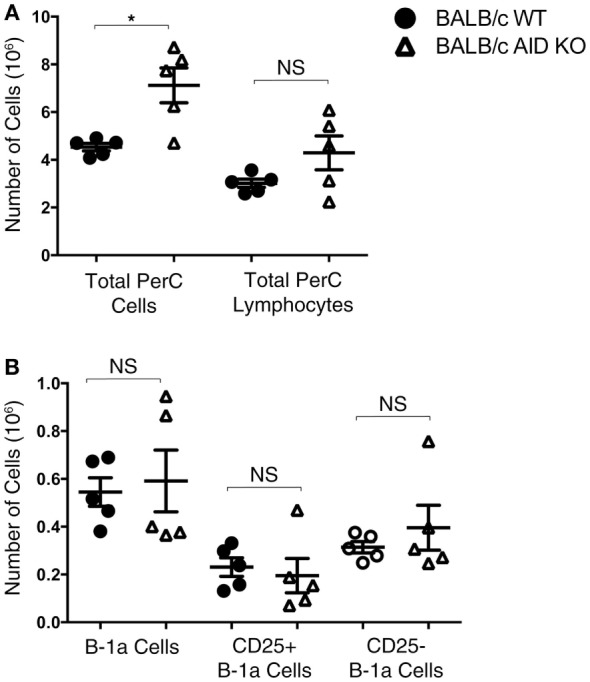
Number of B-1a cells in activation-induced cytidine deaminase (AID) knock out (KO) mice. Peritoneal washout cells were obtained from 3-month-old wild-type (WT) and AID KO mice on the BALB/c-ByJ background. **(A)** The total number of peritoneal cells and the total number of lymphocytes (based on the lymphocyte gate) are shown. **(B)** Peritoneal washout cells were stained with anti-B220-pCP-Cy5.5, anti-CD5-Alexa 647, anti-CD25-PE, and CD23-PE-Cy7. Based on staining for B-1a (B220^lo^CD5^+^), CD25^+^ B-1a (B220^lo^CD5^+^CD25^+^), and CD25^−^ B-1a (B220^lo^CD5^+^CD25^−^), the absolute number of each subset is shown. Results are shown as means of five independent mice, along with lines indicating the SEMs.

Mice lacking the AID gene on the BALB/c background were assessed for numbers of total peritoneal cells, total peritoneal lymphocytes, B-1a cells, CD25^+^ B-1a cells, and CD25^−^ B-1a cells. There was no significant difference in the total number of peritoneal lymphocytes in AID KO mice (4.3 × 10^6^ ± 0.71) compared to that in WT mice (3.0 × 10^6^ ± 0.17) (Figure 2A), although the total number of cells in the peritoneal cavities of AID KO mice was greater than the number in WT mice, presumably due to differences in a non-lymphoid population, such as myeloid cells. Next, the total numbers of B-1a, CD25^+^ B-1a, and CD25^−^ B-1a cells were assessed in WT and AID KO mice. The results demonstrated that there is no significant difference in the total numbers of peritoneal B-1a, CD25^+^ B-1a, or CD25^−^ B-1a cells from AID KO mice compared to those in WT controls (Figure 2B). Thus, AID does not appear to be required for the development of early appearing CD25^+^ or CD25^−^ B-1a cells.

### AID Impairs BM B-1a Cell Development

**Figure 3:**
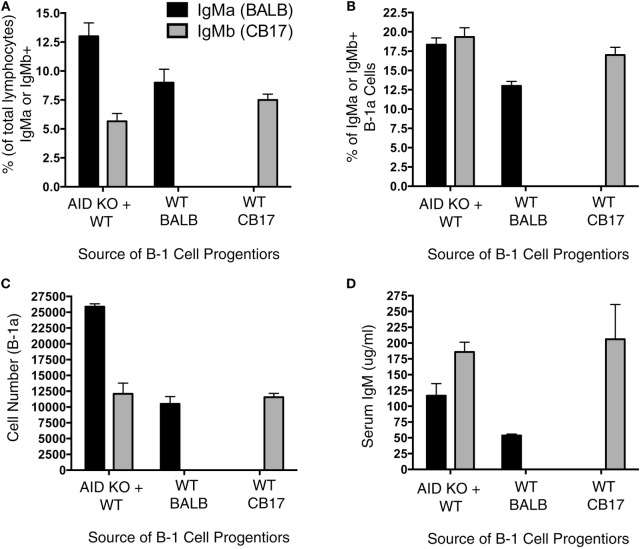
*Aicda* impairs B-1a cell development. Allotype mixed chimeras were set up by injecting (i.v.): (1) 600,000 B-1 cell-specific progenitors (Lin^−^B220^lo/−^CD19^+^AA4.1^+^) obtained from activation-induced cytidine deaminase (AID) knock out (KO)-BALB/c bone marrow (BM) (IgM^a^) along with 600,000 B-1 cell-specific progenitors from wild-type (WT)-CB17 BM (IgM^b^); (2) 600,000 B-1 cell-specific progenitors obtained from WT-BALB/c BM (IgM^a^); or (3) 600,000 B-1 cell-specific progenitors from WT-CB17 BM (IgM^b^) into CB17-SCID recipients. Six weeks after the transfer, peritoneal cells were collected for the flow analysis. **(A)** The percent of live lymphocytes positive for IgM^a^ (black bars) or IgM^b^ (gray bars) in the collected washout cells was assessed. **(B)** The percent of live lymphocytes that phenotyped as IgM^a^ (black bars) or IgM^b^ (gray bars) B-1a cells in the collected washout cells was assessed. **(C)** The total number of peritoneal B-1a cells derived from BALB/c (IgM^a^, black bars) or CB17 (IgM^b^, gray bars) mice was assessed.

It has been previously shown that *Aicda* deficiency impairs B-cell development (13); however, it is unknown whether this effect extends to B-1a cell development. To directly test the extent to which *Aicda* affects B-1a cell development, we set up a mixed chimera system. Figure 3A illustrates the experimental design, which involved adoptive transfer of B-1 cell-specific progenitors (Lin^−^B220^lo/−^CD19^+^AA4.1^+^) obtained from the BM of AID KO mice and WT mice. Three groups of chimera mice were set up: (1) SCID mice were injected with 600,000 B-1-specific progenitors from the BM of BALB/c AID KO mice plus 600,000 B-1-specific progenitors from the BM of CB17 WT mice; (2) SCID mice were injected with 600,000 B-1-specific progenitors from the BM of BALB/c AID KO mice; and (3) SCID mice were injected with 600,000 B-1-specific progenitors from the BM of CB17 WT mice. Allotypic differences between BALB/c-ByJ (IgM^a^) and CB17 (IgM^b^) mice were used to assess the individual contributions of WT (IgM^b^)- and AID KO (IgM^a^)-derived B-1a cells to the B-1a cell pool.

We first examined the percent of total lymphocytes in the peritoneal washouts that were either IgM^a+^ or IgM^b+^. Interestingly, in the chimera mice receiving B-1 cell progenitors from both AID KO and WT BM, there were more AID KO-derived (IgM^a+^) B cells than WT-derived (IgM^b+^) B cells (*p* = 0.01) (Figure 3A). On the other hand, there was no difference in the percent of IgM^a+^ or IgM^b+^ lymphocytes that were peritoneal B-1a cells (Figure 3B) in mice receiving B-1 cell progenitors from both AID KO and WT BM. Thus, the total number of peritoneal B-1a cells derived from WT BM B-1 cell progenitors was significantly lower than those derived from AID KO BM B-1 cell progenitors (*p* = 0.01) (Figure 3C). As a control, BM B-1 cell progenitors from WT mice or AID KO mice were transferred alone into SCID recipients to detect any differences in reconstitution when the progenitors from these two sources (BALB/c or CD17 mice) were transferred alone. The results of the single transfers demonstrated no significant differences in the overall reconstitution of WT or AID KO mouse B-1 cell progenitors when transferred individually (Figures 3A–C). Together, these results demonstrate that *Aicda* inhibits the development of B-1a cells from BM B-1 cell progenitors in a competitive environment.

## Discussion

We found that B-1a cells, particularly CD25^+^ B-1a cells, express *Aicda*. This *Aicda* expression occurs in the absence of intentional stimulation and without participation in GCs. Our results support the findings of Herzenberg and colleagues regarding the loss of somatic mutation and isotype switching from B-1 cell immunoglobulin in the absence of AID (16). Thus, we have extended previously reported functional results by directly demonstrating that WT B-1a cells express *Aicda*, and we have shown that *Aicda* expression is concentrated within the CD25^+^ B-1a cell subpopulation.

Recent studies have utilized AID reporter constructs to identify memory B cells that developed in GCs (18–20). Our results inject a note of caution regarding the interpretation of these kinds of experiments by showing that some mature B cells, specifically CD25^+^ B-1a cells, express *Aicda* and thus could register as reporter-positive despite not having resided in a GC. Further complicating this issue is the recent evidence that some B-1 cells may themselves be memory B cells (21). It is clear from the work reported here and by others that further study will be needed to tease out the extent to which *Aicda* expression marks naïve B-1a as well as memory B-2 cells and/or marks memory B cells regardless of whether they are B-1a or B-2.

We previously found that about 20% of peritoneal B-1a cells express CD25, a component of the high-affinity IL-2 receptor, and CD25^+^ B-1a cells express increased levels of activated signaling intermediates (4). However, these B-1a cells lack expression of CD122 and are not responsive to IL-2 (4). In some systems, AID expression appears to impact viability. Immature murine B cells express AID and AID-deficient immature B cells are more resistant to apoptosis than immature B cells that express AID (15). Moreover, AID-deficient GC B cells are more resistant to apoptosis (22). These data suggest a role for AID in regulating cell viability. Along these lines, we found more B-1 cells in the peritoneal cavities of SCID mice after reconstitution with AID KO B-1 progenitor cells, and this was accentuated in BM chimeras wherein competition exists between WT and KO B-1 cell progenitors. Thus, AID is likely involved in the development and/or viability of B-1a cells.

The phenotype of mouse-like human B-1 cells has recently been redefined (23) from its previous focus on CD5 (24), an unreliable marker for B-1 cells in *Homo sapiens* and other species (25–27). It has been reported that AID-deficient hyper IgM syndrome patients are prone to develop autoimmune or inflammatory diseases such as diabetes mellitus, polyarthritis, autoimmune hepatitis, hemolytic anemia, and immune thrombocytopenia (28, 29). Our results suggest that B-1 cells should be considered as the pathogenesis of AID-deficient autoimmunity is probed.

## Author Contributions

HK, NH, and JT designed and performed the research, analyzed and interpreted data, and wrote the manuscript; TR interpreted data and wrote the manuscript.

## Conflict of Interest Statement

The authors declare that the research was conducted in the absence of any commercial or financial relationships that could be construed as a potential conflict of interest.

## Supplementary Material

Figure S1 *Aicda* gene expression in B cells by Taqman assay. Peritoneal washout cells and spleen cells were obtained from 3-month-old BALB/c-ByJ mice, immunofluorescently stained, and sorted for peritoneal B-1a (B220^lo^CD5^+^), CD25^+^ B-1a (B220^lo^CD5^+^CD25^+^), CD25^−^ B-1a (B220^lo^CD5^+^CD25^−^), splenic B2 (B220^+^CD5^−^CD23^+^), and GC (B220^+^/GL-7^+^/PNA^high^) cells, as shown in Figure 1. RNA was prepared from each sort-purified B cell subset and reverse transcribed. The level of *Aicda* relative to actin was determined by real-time PCR (Taqman) with the primers described in Section “Materials and Methods.” The means of three independent experiments are shown, along with lines indicating SEMs.

Figure S2 *Aicda* gene expression in C57BL/6 B cells. Peritoneal washout cells and spleen cells were obtained from 3-month-old C57BL/6J mice, immunofluorescently stained, and sorted for peritoneal B-1a (B220^lo^CD5^+^), CD25^+^ B-1a (B220^lo^CD5^+^CD25^+^), CD25^−^ B-1a (B220^lo^CD5^+^CD25^−^), splenic B2 (B220^+^CD5^−^CD23^+^), and GC (B220^+^/GL-7^+^/PNA^high^) cells, as shown in Figure 1. RNA was prepared from each sort-purified B cell subset and reverse transcribed. The level of *Acida* relative to β_2_-microglobulin was determined by real-time PCR (SYBR Green) with the primers described in Section “Materials and Methods.” The means of three independent experiments are shown in **(A)**, along with lines indicating SEMs. The level of *Aicda* relative to actin was determined by real-time PCR (Taqman) with the primers described in Section “Materials and Methods.” The means of three independent experiments are shown in **(B)**, along with lines indicating SEMs.
