# Supplementary figures and images for: CD25+ B-1a Cells Express Aicda
Source: Front Immunol. 2017 Jun 20;8:672. doi: 10.3389/fimmu.2017.00672 (PMC5477345; doi:10.3389/fimmu.2017.00672)

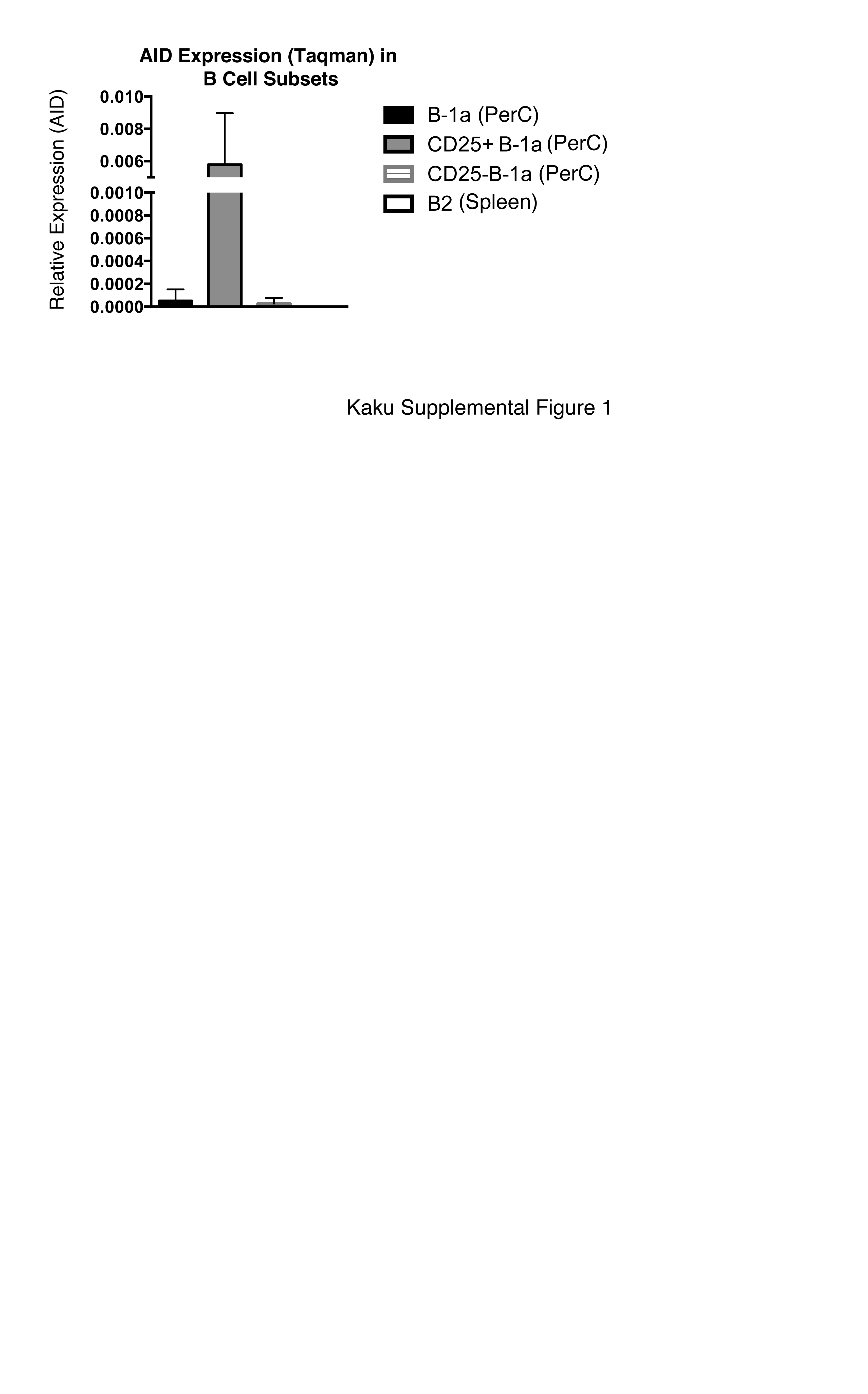

Supplement: Figure S1 — Aicda gene expression in B cells by Taqman assay. Peritoneal washout cells and spleen cells were obtained from 3-month-old BALB/c-ByJ mice, immunofluorescently stained, and sorted for peritoneal B-1a (B220loCD5+), CD25+ B-1a (B220loCD5+CD25+), CD25− B-1a (B220loCD5+CD25−), splenic B2 (B220+CD5−CD23+), and GC (B220+/GL-7+/PNAhigh) cells, as shown in Figure 1. RNA was prepared from each sort-purified B cell subset and reverse transcribed. The level of Aicda relative to actin was determined by real-time PCR (Taqman) with the primers described in Section “Materials and Methods.” The means of three independent experiments are shown, along with lines indicating SEMs. [file image_1.tif]

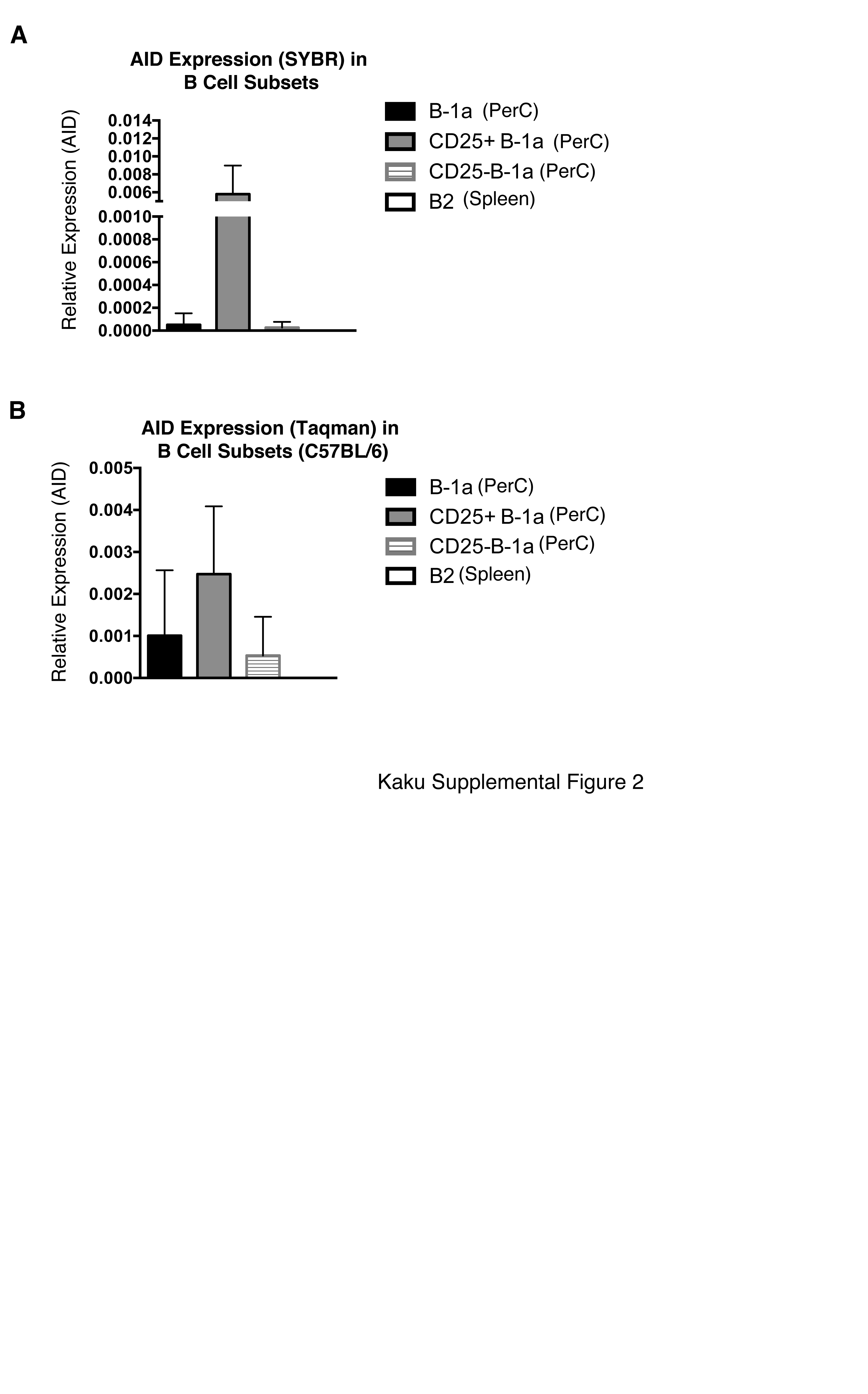

Supplement: Figure S2 — Aicda gene expression in C57BL/6 B cells. Peritoneal washout cells and spleen cells were obtained from 3-month-old C57BL/6J mice, immunofluorescently stained, and sorted for peritoneal B-1a (B220loCD5+), CD25+ B-1a (B220loCD5+CD25+), CD25− B-1a (B220loCD5+CD25−), splenic B2 (B220+CD5−CD23+), and GC (B220+/GL-7+/PNAhigh) cells, as shown in Figure 1. RNA was prepared from each sort-purified B cell subset and reverse transcribed. The level of Acida relative to β2-microglobulin was determined by real-time PCR (SYBR Green) with the primers described in Section “Materials and Methods.” The means of three independent experiments are shown in (A), along with lines indicating SEMs. The level of Aicda relative to actin was determined by real-time PCR (Taqman) with the primers described in Section “Materials and Methods.” The means of three independent experiments are shown in (B), along with lines indicating SEMs. [file image_2.tif]
